# Anatomical variations of the atlas arches: prevalence assessment, systematic review and proposition for an updated classification system

**DOI:** 10.3389/fnins.2024.1348066

**Published:** 2024-02-28

**Authors:** Gloria P. Baena-Caldas, Juan F. Mier-García, Dylan P. Griswold, Adriana M. Herrera-Rubio, Ximara Peckham

**Affiliations:** ^1^Department of Pathology, SUNY Downstate Health Science University, Brooklyn, NY, United States; ^2^Department of Morphology, Biomedical Sciences School, Division of Health Sciences, Universidad del Valle, Cali, Colombia; ^3^School of Dentistry, Division of Health Sciences, Universidad del Valle, Cali, Colombia; ^4^Section of Neurosurgery, Division of Health Sciences, Universidad del Valle, Cali, Colombia; ^5^Department of Neurosurgery, Oxford University Hospitals NHS Foundation Trust, Oxford, United Kingdom; ^6^Stanford School of Medicine, Stanford, CA, United States; ^7^Department of Clinical Neurosciences, University of Cambridge, Cambridge, United Kingdom; ^8^NIHR Global Health Research Group on Acquired Brain and Spine Injury (ABSI), Department of Neurosurgery, University of Cambridge, Cambridge, United Kingdom; ^9^Division of Life Sciences, Long Island University, Brooklyn, NY, United States

**Keywords:** atlas (C1 vertebra), anatomical variation, vertebral arch, cone-beam computed tomography (CBCT), congenital abnormalities, cervical instability

## Abstract

**Objective and background:**

This study focuses on the atlas, a pivotal component of the craniovertebral junction, bridging the cranium and spinal column. Notably, variations in its arches are documented globally, necessitating a thorough assessment and categorization due to their significant implications in clinical, diagnostic, functional, and therapeutic contexts. The primary objective is to ascertain the frequency of these anatomical deviations in the atlas arches among a Colombian cohort using cone-beam computed tomography (CBCT).

**Methodology:**

Employing a descriptive, cross-sectional approach, this research scrutinizes the structural intricacies of the atlas arches in CBCT scans. Analytical parameters included sex distribution and the nature of anatomical deviations as per Currarino’s classification. Statistical analyses were conducted to identify significant differences, including descriptive statistics and Chi-square tests. A systematic review of the literature was conducted in order to enhance the current Currarino’s classification.

**Results:**

The study examined 839 CBCT images, with a nearly equal sex distribution (49.7% female, 50.3% male). Anatomical variations were identified in 26 instances (3%), displaying a higher incidence in females (X2 [(1, *N* = 839) = 4.0933, *p* = 0.0430]). The most prevalent variation was Type A (2.5%), followed by Type B (0.4%), and Type G (0.2%) without documenting any other variation. The systematic review yielded 7 studies. A novel classification system for these variations is proposed, considering global prevalence data in the cervical region.

**Conclusion:**

The study highlights a statistically significant predominance of Type A variations in the female subset. Given the critical nature of the craniovertebral junction and supporting evidence, it recommends an amendment to Currarino’s classification to better reflect these clinical observations. A thorough study of anatomical variations of the upper cervical spine is relevant as they can impact important functional aspects such as mobility as well as stability. Considering the intricate anatomy of this area and the pivotal function of the atlas, accurately categorizing the variations of its arches is crucial for clinical practice. This classification aids in diagnosis, surgical planning, preventing iatrogenic incidents, and designing rehabilitation strategies.

## Introduction

This study centers on the atlas, the foremost cervical vertebra with a critical anatomical and functional position (Swartz et al., 2005). The atlas is distinguished by its unique structure, consisting of anterior and posterior arches and two lateral masses, each featuring a transverse foramen (Standring and Gray, 2021; Lokanathan et al., 2022). This configuration establishes a conduit for essential neurological and vascular elements, including the cervical segment of the spinal cord, meninges, cerebrospinal fluid, suboccipital nerve, spinal root of the accessory nerve, and the vertebral arteries and veins (Dubey et al., 2023). Moreover, the atlas is instrumental in supporting the head and facilitating motion and stability within the craniovertebral region, through its articulatory surfaces and connections to various ligaments and muscles (Cattrysse et al., 2016). Multiple anatomical variations of the atlas have been described such as accessory transverse foramina, ponticulus posticus and alterations in the fusion of its arches (Macrì et al., 2023; Ogut et al., 2023).

Embryological and congenital processes are key in explaining the diverse anatomical variations observed in the atlas (Choi et al., 2011; Piatt Jr and Grissom, 2011). These variations are multifaceted and predominantly involve alterations in the fusion of the posterior arch or its partial or complete absence, as delineated by Currarino et al. (1994), Geist et al. (2014), Kim (2015), and Hinai et al. (2021). While deviations in the anterior arch’s structure are less frequently documented, they are equally significant in terms of structural impact (Martirosyan et al., 2011; Guenkel et al., 2013; Karavelioglu et al., 2014).

Currarino’s classification categorizes these variations into distinct types: Type A signifies a failure in the posterior midline fusion of the hemiarches; Type B represents a unilateral posterior cleft; Type C indicates a bilateral posterior cleft; Type D denotes a partial absence of the posterior arch with a residual posterior tubercle; and Type E encapsulates a complete absence of the posterior arch (Currarino et al., 1994). Notably, congenital defects of the anterior arch were not initially included in Currarino’s framework; however, subsequent studies have identified instances of a cleft in the anterior arch accompanied by a partial or total absence of the posterior arch, termed “bipartite atlas” (Weng et al., 2010; Guenkel et al., 2013; Ulusoy et al., 2017).

Congenital anomalies in the atlas can precipitate instability in the craniovertebral junction and cervical spine, particularly in pediatric cases (Pasku et al., 2007; Chau et al., 2009; Chung et al., 2010; Weng et al., 2010; Park et al., 2011). These anomalies often present diagnostic challenges, as they can be mistakenly identified as fractures, subluxations, or osteolysis, thus underscoring the necessity for comprehensive assessment and diagnosis to inform appropriate therapeutic strategies (Harrop et al., 2008; Carr et al., 2012; Ulusoy et al., 2017).

In light of these considerations, this study aims to evaluate the prevalence of anatomical variations in the anterior and posterior arches of the atlas using Cone-Beam Computed Tomography (CBCT) scans from a Colombian population sample.

## Materials and methods

This research adopted a descriptive, observational, retrospective, and cross-sectional methodological framework. The CBCT images utilized originated from an imaging center in Cali, Colombia, primarily intended for diagnosing and treating various facial and dental conditions, not specifically for this study. During the analysis by a seasoned radiologist, incidental anomalies in the cervical area were observed in these images. The high-resolution capability of CBCT for facial, dental, and cervical regions prompted the undertaking of an extensive analysis of a larger sample. The selection of CBCT in the analysis of anatomical variations is convenient as it has been previously shown that CBCT exhibits spatial accuracy that is higher at the center of the volume than at the margins (Wan et al., 2021).

The retrospective examination encompassed 870 CBCT images collected from November 2015 to December 2018. A total of 31 images were excluded due to low technical quality or by noise generated by metal. Thus 839 images were analyzed. This investigation formed a segment of a broader project titled “Characterization of anatomical variations observed in tomographic images of the head and neck in two imaging centers in Cali, Colombia,” which received approval from the Human Ethics Committee of Universidad del Valle in Cali, Colombia.

The imaging was executed using an I-CAT Next Generation Cone Beam apparatus. A meticulous review and analysis were conducted by an anatomical specialist with over 15 years of experience in morphological studies, corroborated by an oral maxillofacial radiologist with a 25-year professional background. The I-CAT Vision software, provided by the manufacturer, was employed for image analysis. Acquisition of the images was done in a standardized way to ensure proper alignment. The reference lines were the median sagittal line and a horizontal line between the occlusal plane and the intersection between this line and a vertical line anterior to the mandibular condyles. The analysis of the images was performed with the following criteria: Sharpening of the filter, multiplanar (MPR) navigation, alignment of the median guideline in the coronal and axial planes, and alignment on the sagittal view of the palatal plane with the horizontal guideline between the anterior and posterior nasal spines.

Inclusion criteria were confined to images representing the Colombian population. Exclusion criteria involved images of inferior technical quality or those affected by noise due to metal dental restorations or implants.

### Statistical analysis

The primary variables investigated included sex (male or female) and the complete or partial absence of the atlas’s posterior arch, following Currarino et al.’s classification (Currarino et al., 1994). This included the precise location of the absence (midline, right, or left side), along with defects in the anterior arch.

The descriptive data analysis that was performed is presented using absolute and relative frequencies as nominal variables are used. The Chi-square test was used to assess the statistical disparities between sex and anatomical variations, considering a value of *p* ≤ 0.05 as statistically significant. Data analysis was executed using the SPSS software, version 29.

### Systematic review

Furthermore, a systematic literature review was conducted to aggregate existing knowledge on the anatomical deviations of the atlas’s anterior arch.

For the literature search, medical subject headings (MeSH) terms, and related text words were used, with an effort made to account for synonyms, acronyms, plurals, and variations in spelling. A comprehensive search was performed in PubMed, OVID, EMBASE, Web of Science and in the Cochrane Central Register of Controlled Trials from their inception until June 2023, in English or Spanish and without restriction of time.

To control publication bias, in addition to the exhaustive search in the different databases, references of relevant articles not initially identified were manually scanned.

Manuscripts that met the following criteria were considered eligible for inclusion: (1) of human adults with anatomical variations on the atlas arches.

*In vitro* studies, reviews, abstracts, and conference proceedings were excluded. Additionally, manuscripts were excluded if included (1) patients with history or diagnosis of genetic syndromes or (2) pediatric population.

All the reviewers independently screened the titles and abstracts of all the retrieved records. Later, the relevant studies were reviewed in full text separately, and either included or excluded based on the eligibility criteria. In the case of unresolved discordance, a corresponding third author would adjudicate.

All the reviewers separately extracted data in 3 Excel sheets, crosschecking them against each other and the source material. The following data was extracted: Study objective, country where the study was conducted, sex and age, type of anterior arch anomaly, additional anomalies and other relevant data.

We finally performed a narrative synthesis of our review, describing clinical and methodological characteristics of the included studies.

This facilitated the development of a supplementary classification system to augment that proposed by Currarino et al. (1994).

## Results

A total of 839 CBCT images that met the inclusion criteria were analyzed. The age range of the individuals spanned from 18 to 93 years, averaging at 55 years. Table 1 delineates the observed variations in the atlas arches, segmented by sex.

**Table 1:** Sex-based distribution of anatomical variations in the atlas arches.

Sex	Anatomical variations of the arches of the atlas	
	Absent (*n* = 813; 96.9%)	Present (*n* = 26; 3.1%)
Males (*n* = 422; 50.3%)	414 (51%)	8 (31%)
Females (*n* = 417; 49.7%)	399 (49%)	18 (69%)

Among the analyzed images, 3% (*n* = 26) exhibited at least one variation in the arches. Notably, a higher prevalence was found in females, accounting for 69% (*n* = 18) of these variations, compared to 31% (*n* = 8) in males. This disparity was statistically significant [X2 (1, *N* = 839) = 4.0933, *p* = 0.0430].

**Figure 1:**
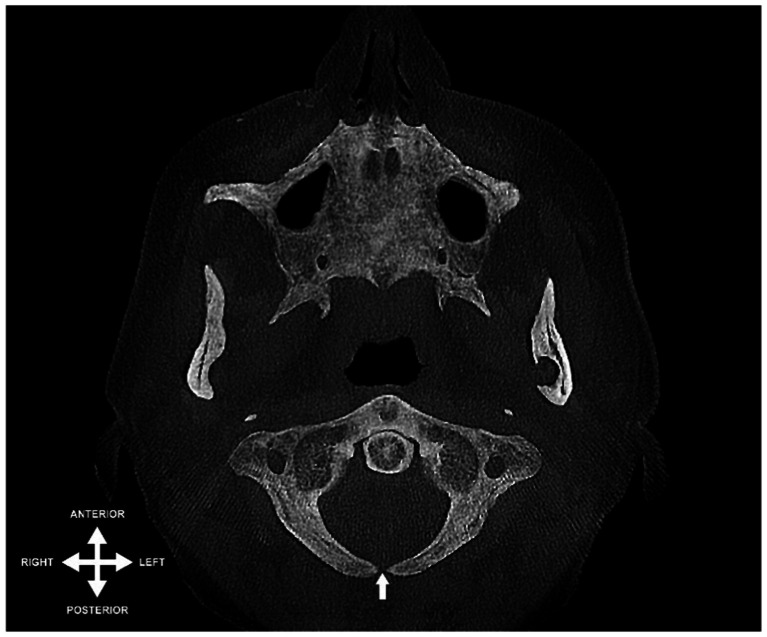
Axial view of a CBCT scan depicting midline cleft in the posterior arch of the atlas (indicated by arrow) – an instance of Currarino’s type A variation.

The predominant variation identified was a median cleft in the posterior arch, aligning with Currarino’s Type A classification. This was evident in 21 cases (2.5%), predominantly in females (71%, *n* = 15) compared to males (29%, *n* = 6). Figure 1 illustrates a representative example of this finding. Additionally, a lateral cleft in the posterior arch, corresponding to Currarino’s Type B, was observed in 3 instances (0.4%), with a distribution of 2 females (67%) and 1 male (33%), all presenting on the left side.

**Figure 2:**
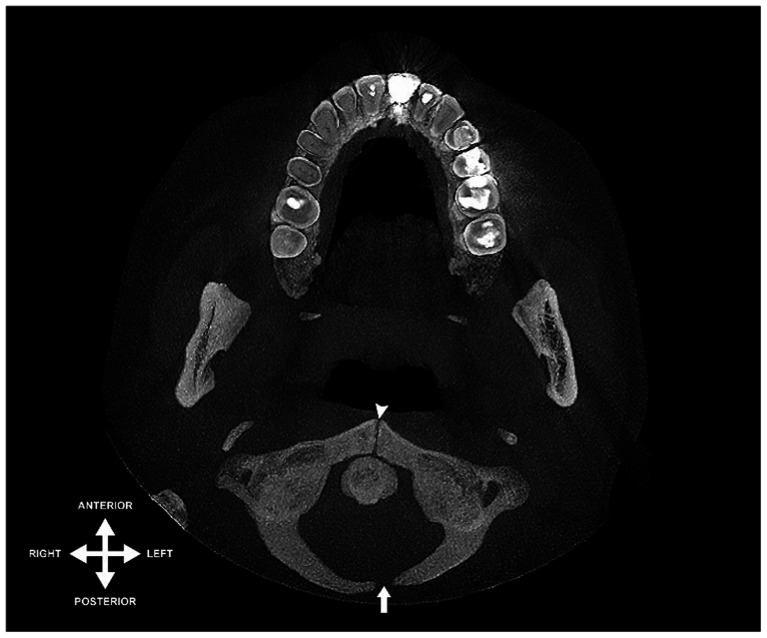
Axial view of CBCT scan illustrating a bipartite atlas: midline cleft in anterior arch (indicated by arrowhead) and left lateral partial absence in posterior arch (indicated by arrow).

Furthermore, two instances (0.2%) of a bipartite atlas were recorded, featuring a median fissure in the anterior arch and a left lateral partial cleft in the posterior arch, equally divided between male and female subjects. Figure 2 provides an example of this particular anatomical variant.

**Figure 3:**
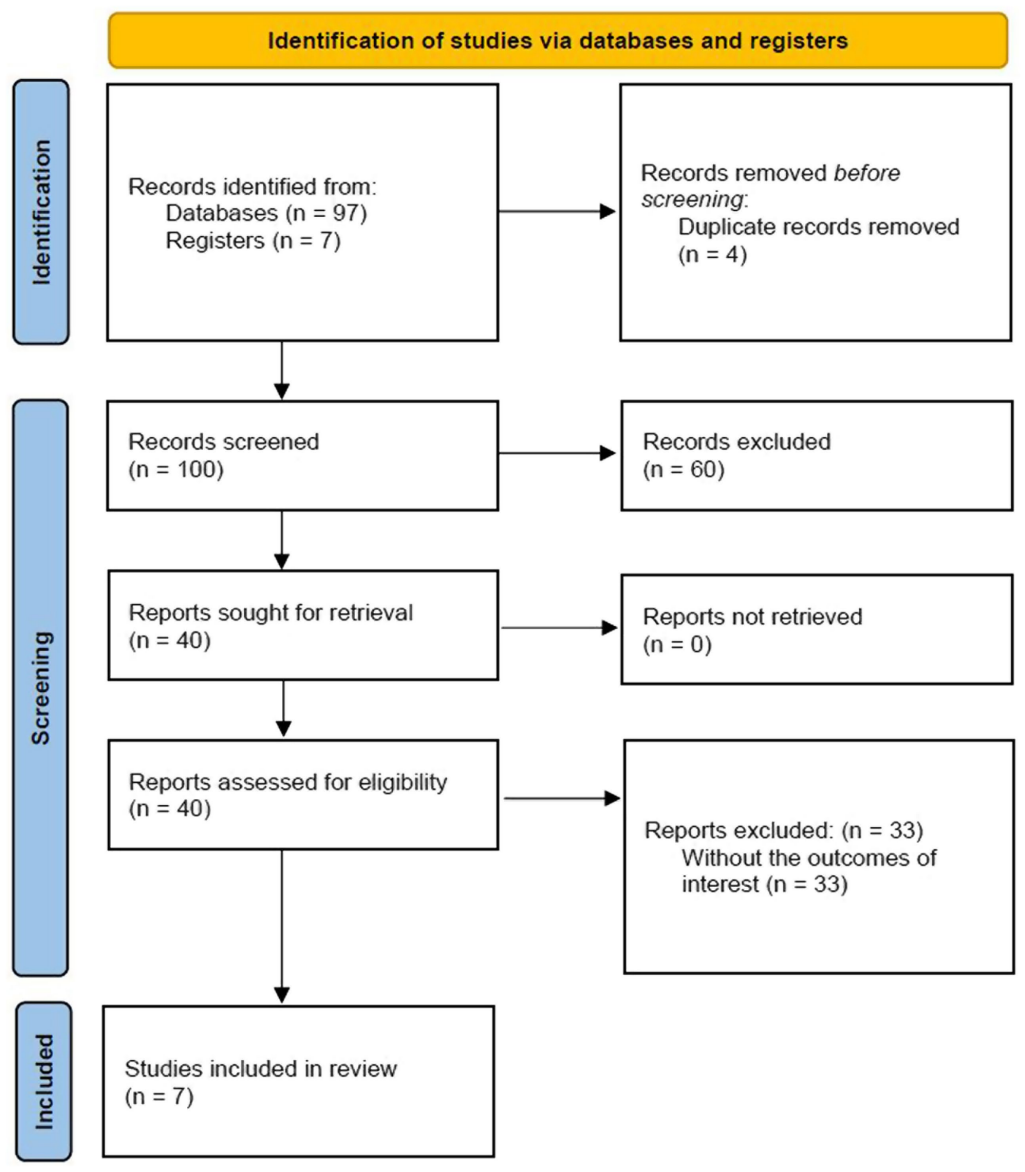
PRISMA diagram.

Utilizing the search strategy described above, 97 records were found. Seven citations were manually scanned. After the removal of duplicate citations, a total of 100 studies were subsequently reviewed. Review of titles and abstracts excluded 60 citations based on their failure to meet inclusion criteria. Forty studies were deemed potentially suitable for inclusion and reviewed in full text. Of these 33 did not include the outcome of interest describing different anomalies of the atlas. Seven articles were finally included for data extraction. The PRISMA flow diagram is shown in Figure 3.

The 7 included studies were published between 1986 and 2018, and conducted in Canada (Piatt Jr and Grissom, 2011), China (Choi et al., 2011), India (Currarino et al., 1994), South Korea (Geist et al., 2014; Kim, 2015), Turkey (Hinai et al., 2021) and the United States of America (Martirosyan et al., 2011), with a total of 8 patients. All of the included records were case reports. A summary of the main characteristics of the included records with patient demographics is presented in Table 2.

**Table 2:** Comprehensive review of individual case studies from literature: anomalies of the anterior arch of the atlas.

Author and country of origin	Sex and age (years)	Type of anterior arch anomaly	Diagnostic methods used	Additional anomalies	Associated symptoms and other data	Treatment or management strategies	Follow-up information	Outcome or prognosis
Choi et al. (2011), Korea	Male, 46	Midline cleft within the anterior arch of the atlas.	X rays, CT scan, MRI, Fluoroscopy.	Bilateral bony defects of the lateral aspects of the posterior arch of the atlas. Increased cord signal at the axis level on the T2-weighted sagittal image without compression of the cord. Bilateral bony defects of the lateral aspects of the atlas posterior arch. The most dorsal part of the posterior arch was preserved. Anterior movement of the bony remnant of the posterior atlas was independent of the atlas anterior arch causing compression of the spinal cord during extension.	Two-month history of tremor and hyperesthesia of the lower extremities after experiencing a minor head trauma. Quadriplegia for about 2 weeks after that trauma. Difficulty with voiding and defecation. X-rays showed no instability of the occipitoatlantoaxial area. Movement of a bony remnant of the posterior arch. On neurological examination: Normal motor function. Decreased light touch in lower extremities and right hand. Positive Lhermitte’s sign. Saddle anesthesia. Decreased anal sphincter tone.	Initial conservative management, posterior excision of the remnant posterior tubercle via suboccipital midline approach. No occipitocervical fusion was performed.	Gradual improvement of motor weakness. A 12 month follow-up radiograph showed no instability of the occipitocervical complex.	6 weeks after the operation, there was significant improvement of his neurological symptoms. There was no neck pain, tremor of lower extremities, Lhermitte’s sign or hyperesthesia below the T12 dermatome. However, the low anal sphincter tone remained.
Mace and Holliday (1986), United States	Male, 19	Anterior cleft	X rays, CT scan	Thickening of the prevertebral soft tissues anterior to C1.	Neck pain and stiffness. History of trauma. No neurological deficit.	Conservative treatment.	None	The patient recovered and did well.
Mace and Holliday (1986), United States	Male, 13	Anterior cleft	X rays, CT scan, MRI, Fluoroscopy.	Thickening of the prevertebral soft tissues anterior to C1. No posterior arch defect.	Neck pain and headache. History of trauma. No neurological deficit	First event of head trauma: Conservative treatment. Several months after the initial visit, he suffered a new head trauma while wrestling. Tjis time, the neurological examination showed “decreased sensation in the left arm. No motor deficit. The patient was diagnosed with cervical instability. Fusion of C1 and C2 was performed.	None	The patient recovered and did well.
van der Velde et al. (1997), Canada	Male, 19	Anterior cleft - Spondyloschisis (non-union) of the anterior arch. Widening of the lateral masses and a vertical lucency overlying the odontoid.	X rays, CT scan	Not described.	Cervical active ranges of motion moderately decreased, acute pain elicited by left lateral flexion, and extension, and by passive extension combined with rotation and axial compression of the head. Pain was also elicited by palpation of the sternocleidomastoid, scalenus and trapezius muscles and by palpation of joints from CI to C4. History of acute neck injury sustained three days earlier during a high school football game. Neurological examination was normal.	Initial conservative treatment: Ice, rest, electrotherapy, soft tissue therapy, spinal manipulation to the mid-cervical spine, and exercise. Six weeks after the injury. He was allowed to resume playing.	None	Not described.
Kwon et al. (2009), Korea	Female, 59	Cleft	CT scan	C6-7 fusion	Neck pain, arm pain.	Not described.	None	Not described.
Garg et al. (2004), India	Male, 16	Anterior arch aplasia associated with os odontoideum.	X rays, CT scan, MRI.	Ventral displacement of the atlas over the axis on flexion, which reduced on extension.	Weakness and numbness of all four limbs.	Transoral odontoid-ectomy and posterior fixation of occiput with C2–C3 spi-nous processes was performed.	The patient had significant improvement over next 3 months.	The patient had significant improvement over next 3 months.
He and Xu (2012), China	Female, 46	Anterior midline cleft of the atlas.	X-rays, CT scan, MRI.	Odontoid tip ahead of the anterior arch of the atlas.	6-day history of right limbs numb and left upper and lower extremities paraparesis (Frankel D)	The patient underwent Gardner–Wells tong traction and surgery of occipitocervical fusion with autoge-nous iliac bone graft.	Atlantoaxial reduction was confirmed with fluo-roscopic X-ray evaluation by bed, her neurological deficit was resolved from Frankel D to E, and numb of right limbs completely disappeared 6 days postoperation	No instability has been observed during 2-year follow-up.
Ulusoy et al. (2017), Turkey	Female, 56	Anterior arch cleft.	CT scan (Lokanathan et al., 2022), MRI.	Anteriorly subluxed right atlantoaxial joint.	Physical examination revealed no pathological findings with a full range of cervical movement and a normal neurological status.	Conservatively	Asymptomatic	Asymptomatic

## Discussion

The prevalence of anatomical variations in at least one of the two arches of the atlas, as reported in previous literature, ranges between 0.074 and 5.16%, based on findings from both cadaveric and imaging studies (Senoglu et al., 2007; Hyun et al., 2018; Sanchis-Gimeno et al., 2018; Hinai et al., 2021). These statistics are in alignment with the results of the current study. Furthermore, Currarino’s Type A variations are identified as the most commonly reported anatomical anomaly of the atlas, with existing literature indicating a prevalence of 1.5–5% (Guenkel et al., 2013; Geist et al., 2014; Souza et al., 2014; Ulusoy et al., 2017; Hyun et al., 2018; Cossu et al., 2019; Tsoucalas et al., 2020). This prevalence rate is corroborated by the 2.5% observed in the present study.

A notable aspect of this study is the sex-related differences in these variations. Females exhibited a higher rate of variations (2.15%) compared to males (0.95%), corroborating findings from Hyun et al., who noted similar trends in their extensive CT scan analysis (Hyun et al., 2018). The occurrence of a bipartite atlas, while rare, was observed in our study, aligning with the low prevalence rates (0.2–0.3%) reported in previous studies by Currarino et al. (1994) and Guenkel et al. (2013). However, an isolated cleft of the anterior arch, a very rare condition, was not detected in our series.

In light of our findings, as we undertook a comprehensive evaluation of the existing literature to more thoroughly delineate the reported prevalence of the defects in the arches of the atlas. This involved an extensive review and documentation of their occurrence rates in larger-scale imaging studies, alongside an assessment of the number of individually reported cases in this domain, being this the first work addressing this gap in the knowledge.

In their seminal review focusing on anterior arch defects of the atlas, Karavelioglu et al. (2014). documented a total of seven cases involving congenital anomalies. This included four instances of anterior arch clefts and three cases where the anterior arch was absent (Mace and Holliday, 1986; Hosalkar et al., 2001; Park et al., 2006; Senoglu et al., 2007; Kwon et al., 2009; Thavarajah and McKenna, 2012; Karavelioglu et al., 2014). Notably, three of these cases were associated with a personal history of trauma, and an equal number presented with concurrent defects in the posterior arch. Among these, only one case was observed to have neurological deficits.

Furthermore, the analysis of anterior arch defects revealed three distinct patterns: (a) an isolated cleft of the anterior arch, (b) aplasia encompassing the entire anterior arch, and (c) combined defects of both the anterior and posterior arches. The latter category includes conditions such as anteroposterior spondyloschisis, anteroposterior rachischisis, split atlas, and bipartite atlas (Guenkel et al., 2013; Karavelioglu et al., 2014; Ulusoy et al., 2017).

The embryological development and associated processes of the atlas are pivotal in understanding its anatomical variations (Offiah and Day, 2017). Typically, ossification of the atlas begins in the seventh week of intrauterine life with three primary ossification centers: two lateral and one anterior (Piatt Jr and Grissom, 2011; Offiah and Day, 2017; Ulusoy et al., 2017). The lateral ossification centers are responsible for the formation of the lateral masses and, around the third or fourth year of life, they extend posteriorly to create the posterior arch. The predominant pattern for the ossification of the anterior arch involves a single midline ossification center that expands laterally across cartilaginous tissue, eventually merging with the lateral masses. This process typically occurs between the third and ninth year of life (Garg et al., 2004; Piatt Jr and Grissom, 2011; Ulusoy et al., 2017). In exploring the embryological and developmental aspects of the atlas, we find that typical ossification involves three primary centers emerging around the seventh week of intrauterine life. However, variations in this process can lead to congenital midline clefts, either due to non-fusion of twin ossification centers or the failure of the anterior tubercle’s ossification center to develop.

Choi et al. reported that approximately 2% of individuals exhibit a variation in which a fourth ossification center arises, leading to the formation of a posterior tubercle between the two neural arches (Choi et al., 2011). This event typically occurs around the second year of life (Garg et al., 2004; Ulusoy et al., 2017).

Moreover, there are notable variations in the ossification pattern that can lead to the development of congenital midline clefts. Two primary mechanisms have been identified for this occurrence: (a) the failure of twin ossification centers of the anterior arch to fuse, or (b) the absence of the ossification center at the anterior tubercle, coupled with the failure of the lateral masses’ ossification centers to merge (Garg et al., 2004; Piatt Jr and Grissom, 2011; Ulusoy et al., 2017).

Anatomically, the anterior tubercle of the atlas’s anterior arch serves as a pivotal point of attachment for the anterior longitudinal ligament and the longus coli muscle, while its posterior surface offers an articular surface for the odontoid process of the axis (Yow et al., 2020). Consequently, congenital anomalies that lead to anatomical variations in the atlas or the axis may predispose individuals to instability within the cervical spine (Öğüt et al., 2020). Such instability can potentially result in severe myelopathy, damage to the lower cranial and upper cervical nerves, as well as lesions affecting the vertebral vessels (Martirosyan et al., 2011; Guenkel et al., 2013; Karavelioglu et al., 2014).

Congenital variations in the cervical spine, such as clefts, can be easily misidentified as fractures, subluxations, or instances of osteolysis, necessitating a comprehensive evaluation (Ulusoy et al., 2017). Specifically, clefts of the anterior arch of the atlas may present similarly to Jefferson fractures or vertical fractures of the anterior arches (Harrop et al., 2008). In trauma cases, accurately distinguishing between a congenital anomaly and a fracture line is crucial for determining the appropriate therapeutic approach (Carr et al., 2012).

Fractures are typically characterized by borders with irregularity and, in the context of cervical trauma, the presence of soft tissue edema is not uncommon. This can be contrasted with congenital clefts, which generally exhibit smooth edges and lack involvement of the cortical wall or tissue edema (Karavelioglu et al., 2014; Hinai et al., 2021).

Pediatric populations warrant special consideration, particularly given that children under the age of 3 are at heightened risk for cervical injuries. Furthermore, the potential for misdiagnosis of congenital anomalies is significant in children younger than 8 years, a period during which complete ossification processes may not have fully occurred (Harrop et al., 2008; Carr et al., 2012).

The symptomatology associated with the aforementioned anatomical variations is notably diverse, ranging from pain in the cervical region, glenohumeral joint, and upper extremities, to manifestations such as Lhermitte’s sign (Sagiuchi et al., 2006). More severe neurological deficits, including quadriparesis or quadriplegia, can also occur. However, establishing clinical correlations in this study was challenging due to the absence of detailed clinical information related to the acquired imaging (Currarino et al., 1994; Choi et al., 2011; Karavelioglu et al., 2014; Sanchis-Gimeno et al., 2018).

The full extent of the risk posed by craniovertebral instability and subsequent myelopathy due to anatomical variations of the atlas remains an area requiring further investigation and comprehension. Nevertheless, the importance of accurately recognizing and evaluating these variations cannot be overstated. This is particularly crucial in various surgical interventions targeting the craniovertebral region, as well as in rehabilitation protocols administered by physiotherapists or chiropractors. Additionally, it holds significance in the context of certain sports activities, which can exert considerable forces and pose a risk of injury to the cervical craniovertebral junction (van der Velde et al., 1997).

The occurrence of clefts in the arches of the atlas is of considerable significance in the context of craniovertebral surgeries and surgical cervical arthrodesis procedures. This relevance is particularly pronounced during the fixation of screws in the lateral masses or anterior plates on the atlas. In such surgical interventions, the misplacement of implants presents a serious risk, with potential for severe neurological or vascular complications. Therefore, accurate identification and understanding of these anatomical variations are critical to minimize the risk of such adverse outcomes (Weng et al., 2010; Guenkel et al., 2013).

**Figure 4:**
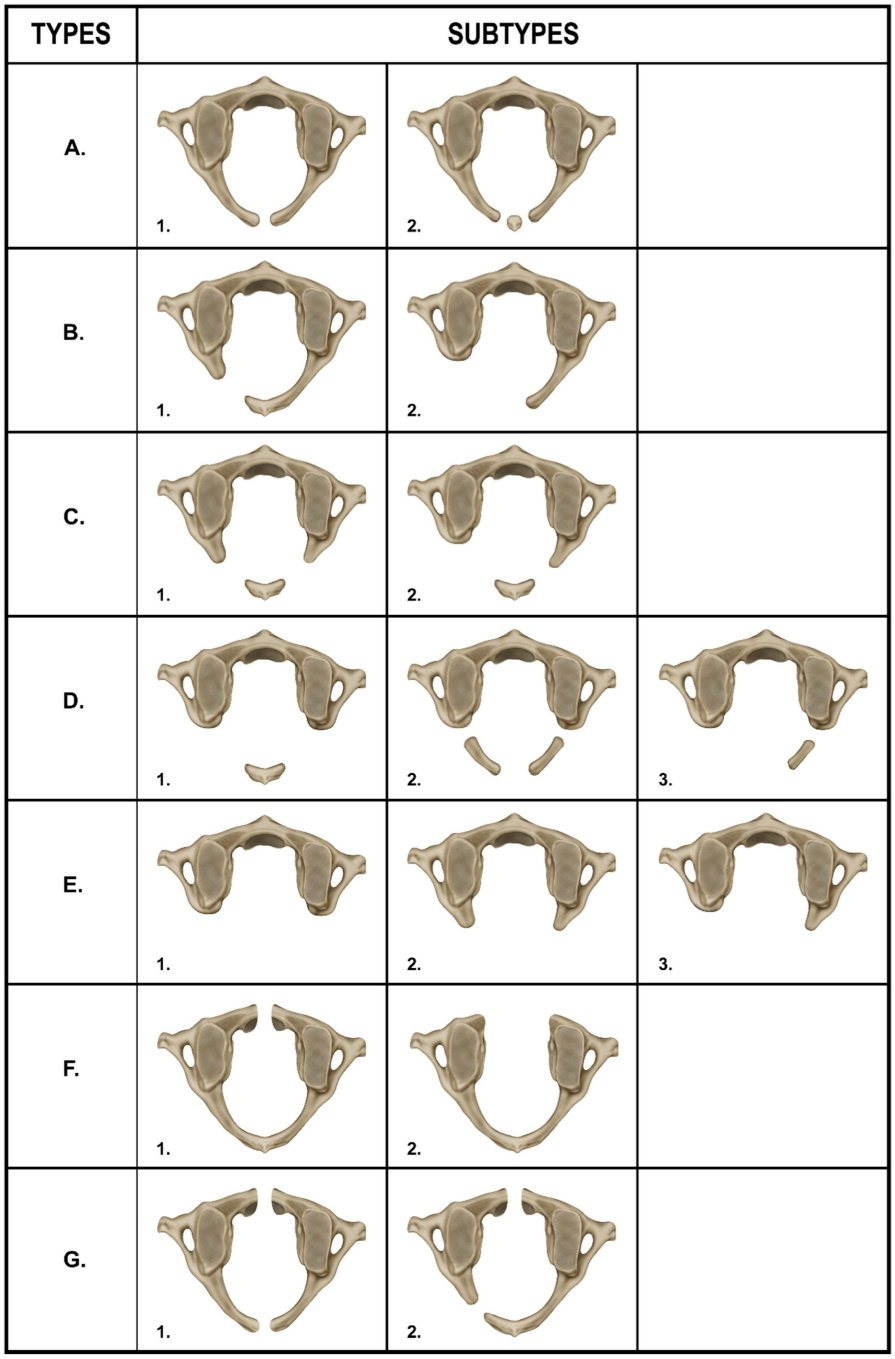
Type A – 1 midline posterior cleft in the atlas, Type A – 2 small ossicle within the midline posterior cleft, Type B – 1 partial unilateral posterior cleft, Type B – 2 complete absence of one of the posterior hemiarches, and absence of the posterior tubercle, Type C – 1 bilateral partial defect in the posterior arch of the atlas with preservation of the posterior tubercle, Type C – 2 complete absence of one posterior hemiarch, partial defect in the other hemiarch, arm and posterior tubercle are preserved, Type D – 1 bilateral complete absence of both posterior hemiarches with a single posterior midline tubercle present, Type D – 2 bilateral cleft in both posterior arms and posterior tubercle absent, Type D – 3 unilateral absence of one posterior hemiarch, cleft of the contralateral arm and posterior tubercle absent, Type E – 1 complete absence of the posterior arch and absence of the posterior tubercle, Type E – 2 partial absence of the both posterior hemiarches and absence of the posterior tubercle, Type E – 3 absence of one of the posterior hemiarches, partial absence of the contralateral arm and posterior tubercle absent, Type F – 1 presence of a midline cleft in the anterior arch of the atlas, Type F – 2 complete absence of the anterior arch, Type G – 1 combined midline defects (clefts) in both the anterior and posterior arches of the atlas (bipartite atlas), Type G – 2 combined defects in the anterior and posterior arches (bipartite atlas) that include a midline cleft in the anterior arch and a unilateral cleft in one of the posterior hemiarches, the posterior tubercle is preserved.

Given the omission of defects of the anterior arch of the atlas in the classification system proposed by Currarino et al. it is proposed to expand this classification by introducing two additional categories, namely Types F and G (Currarino et al., 1994). These new categories, detailed in Table 3 and Figure 4, are suggested in recognition of the existence of these anatomical variations. They underscore the necessity for their thorough radiological, clinical, and surgical evaluation, thereby enhancing awareness and understanding of these specific defects.

**Table 3:** Enhanced Currarino classification: revised typology of atlas arches variations.

Type A	1. The occurrence of a midline cleft in the atlas, which is attributed to the failure of the posterior midline fusion of the two hemiarches. 2. The presence of a small, distinct ossicle within the cleft, arising from the incomplete posterior midline fusion of the two hemiarches.
Type B	1. The presence of a unilateral posterior cleft located in one of the arms of the posterior arch of the atlas. 2. The complete absence of one of the posterior hemiarches, encompassing the posterior tubercle.
Type C	1. A bilateral defect in the posterior arch of the atlas, characterized by the preservation of both posterior arms as well as the posterior tubercle. 2. The complete absence of one posterior hemiarch, coupled with a partial defect in the other hemiarch, yet maintaining the preservation of its arm and the posterior tubercle.
Type D	1. A bilateral and complete absence of both posterior hemiarches of the atlas. This anomaly is typically accompanied by a single, unattached posterior tubercle, often positioned in the midline. 2. A bilateral cleft in both posterior hemiarches, coupled with the absence of the posterior tubercle. 3. The unilateral absence of one posterior hemiarch, cleft of the contralateral arm and cleft of the contralateral arm and absence of the posterior tubercle.
Type E	1. The complete absence of the entire posterior arch of the atlas, which includes the absence of the posterior tubercle. 2. The partial absence of both posterior hemiarches, while both posterior arms are preserved. In this configuration, the posterior tubercle is notably absent. 3. The absence of an entire posterior hemiarch, including the posterior tubercle, with the partial preservation of the contralateral arm.
Type F	1. The presence of a midline cleft in the anterior arch of the atlas. 2. The complete absence of the entire anterior arch.
Type G	1. Combined defects in both the anterior and posterior arches of the atlas, characteristic of a bipartite atlas. This includes a midline cleft in the anterior arch, as well as a midline cleft in the posterior arch. 2. Combined defects in the anterior and posterior arches, indicative of a bipartite atlas, encompassing a midline cleft in the anterior arch and a unilateral cleft in one of the posterior hemiarches, with the preservation of the posterior tubercle.

The present study is unique as it analyses radiological data, complements it with a systematic literature review that includes research articles and individual case reports. Based on our findings we propose a novel classification.

### Limitations

A significant constraint of this study was the absence of clinical histories accompanying the diagnostic imaging. This limitation precluded the possibility of correlating the identified anatomical variations of the atlas with specific clinical manifestations. As a result, the study’s findings are primarily anatomical and cannot be directly linked to patient symptoms or outcomes. This gap highlights the need for future research that integrates clinical data to better understand the implications of these anatomical variations in a clinical context.

The low prevalence of this entities, besides the scarcity of published evidence yielded only case reports as the product of the systematic review.

### Strengths

The relevance of the present study lies in a careful analysis of images combined with a systematic literature review addressing both the existing research articles as well as individual case reports. Interestingly, it was noted that the current classification overlooked prevalent congenital defects of the anterior arch, therefore affecting the proper assessment, diagnosis and therapeutic strategies.

## Conclusion

The study highlights a statistically significant predominance of Type A subtype 1 variations in the female subset, with Type B subtype 1 and Type G subtype 2 being also present, according to our proposed classification. Given the critical nature of the craniovertebral junction and our supporting evidence based on research articles and case reports, we recommend an amendment to Currarino’s classification to better reflect these findings. Considering the pivotal role of the atlas in the cervical region, a proper identification of its variations is relevant in the light of clinical practices including interpretation of symptoms, precise diagnosis, rehabilitation practices, surgical approaches, and minimization of iatrogenic events. This study’s findings highlight the need for ongoing research and education in this domain to ensure patient safety and optimize clinical outcomes.

## Data availability statement

The raw data supporting the conclusions of this article will be made available by the authors, without undue reservation.

## Ethics statement

The studies involving humans were approved by Human Ethics Committee of Universidad del Valle, Cali, Colombia. The studies were conducted in accordance with the local legislation and institutional requirements. The participants provided their written informed consent to participate in this study.

## Author contributions

GB-C: Writing – original draft, Writing – review & editing. JM-G: Writing – original draft, Writing – review & editing. DG: Writing – original draft, Writing – review & editing. AH-R: Writing – original draft, Writing – review & editing. XP: Writing – original draft, Writing – review & editing.
