# Corrigendum: Anatomical variations of the atlas arches: prevalence assessment, systematic review and proposition for an updated classification system

**DOI:** 10.3389/fnins.2024.1418734

**Published:** 2024-05-13

**Authors:** Gloria P. Baena-Caldas, Juan F. Mier-García, Dylan P. Griswold, Adriana M. Herrera-Rubio, Ximara Peckham

**Affiliations:** ^1^Department of Pathology, SUNY Downstate Health Science University, Brooklyn, NY, United States; ^2^Department of Morphology, Biomedical Sciences School, Division of Health Sciences, Universidad del Valle, Cali, Colombia; ^3^School of Dentistry, Division of Health Sciences, Universidad del Valle, Cali, Colombia; ^4^Section of Neurosurgery, Division of Health Sciences, Universidad del Valle, Cali, Colombia; ^5^Department of Neurosurgery, Oxford University Hospitals NHS Foundation Trust, Oxford, United Kingdom; ^6^Stanford School of Medicine, Stanford, CA, United States; ^7^Department of Clinical Neurosciences, University of Cambridge, Cambridge, United Kingdom; ^8^NIHR Global Health Research Group on Acquired Brain and Spine Injury (ABSI), Department of Neurosurgery, University of Cambridge, Cambridge, United Kingdom; ^9^Division of Life Sciences, Long Island University, Brooklyn, NY, United States

**Keywords:** atlas (C1 vertebra), anatomical variation, vertebral arch, cone-beam computed tomography (CBCT), congenital abnormalities, cervical instability

In the published article, there was an error in the caption for **Figure 4** as published. The legend previously stated:

Recommended enhancements to the Currarino classification of atlas arch variations. Type A – 1 midline posterior cleft in the atlas, Type A – 2 small ossicle within the midline posterior cleft, Type B – 1 partial unilateral posterior cleft, Type B – 2 complete absence of one of the posterior hemiarches, Type C – 1 bilateral partial defect in the posterior arch of the atlas with preservation of the posterior tubercle, Type C – 2 complete absence of one posterior hemiarch, partial defect in the other hemiarch, arm and posterior tubercle are preserved, Type D −1 bilateral complete absence of both posterior hemiarches with a single posterior midline tubercle present, Type D – 2 bilateral absence of both posterior arms, absent posterior tubercle, Type D – 3 unilateral absence of one posterior hemiarch, absence of the contralateral arm and posterior tubercle, Type E – 1 complete absence of the posterior arch and absence of the posterior tubercle, Type E – 2 absence of the posterior arch, both posterior arms are preserved with absence of the posterior tubercle, Type E – 3 absence of the posterior hemiarch, absence of the posterior tubercle and the contralateral arm is preserved, Type F – 1 presence of a cleft in the anterior arch of the atlas, Type F – 2 complete absence of the anterior arch, Type G – 1 combined defects (clefts) in both the anterior and posterior arches of the atlas (bipartite atlas), Type G – 2 combined defects in the anterior and posterior arches (bipartite atlas) that include a cleft in the anterior arch and a unilateral cleft in one of the posterior hemiarches. Arm and posterior tubercle are preserved.

The corrected caption appears below:

Type A – 1 midline posterior cleft in the atlas, Type A – 2 small ossicle within the midline posterior cleft, Type B – 1 partial unilateral posterior cleft, Type B – 2 complete absence of one of the posterior hemiarches, and absence of the posterior tubercle, Type C – 1 bilateral partial defect in the posterior arch of the atlas with preservation of the posterior tubercle, Type C – 2 complete absence of one posterior hemiarch, partial defect in the other hemiarch, arm and posterior tubercle are preserved, Type D – 1 bilateral complete absence of both posterior hemiarches with a single posterior midline tubercle present, Type D – 2 bilateral cleft in both posterior arms and posterior tubercle absent, Type D – 3 unilateral absence of one posterior hemiarch, cleft of the contralateral arm and posterior tubercle absent, Type E – 1 complete absence of the posterior arch and absence of the posterior tubercle, Type E – 2 partial absence of the both posterior hemiarches and absence of the posterior tubercle, Type E – 3 absence of one of the posterior hemiarches, partial absence of the contralateral arm and posterior tubercle absent, Type F – 1 presence of a midline cleft in the anterior arch of the atlas, Type F – 2 complete absence of the anterior arch, Type G – 1 combined midline defects (clefts) in both the anterior and posterior arches of the atlas (bipartite atlas), Type G – 2 combined defects in the anterior and posterior arches (bipartite atlas) that include a midline cleft in the anterior arch and a unilateral cleft in one of the posterior hemiarches, the posterior tubercle is preserved.

In the published article, there was also an error in Figure 3 as published. The image for Figure 3 should be the image that was published as Figure 4. In addition, in Figure 3 there were errors in the n numbers provided. “Registers (n = 6)” should be “Registers (n = 7)”. “Records screened (n = 99)” should be “Records screened (n = 100)”. “Reports sought for retrieval (n = 39)” should be “Reports sought for retrieval (n = 40)”. “Reports assessed for eligibility (n = 39)” should be “Reports assessed for eligibility (n = 40)”. “Studies included in review (n = 6)” should be “Studies included in review (n = 7)”. The corrected Figure 3 and its caption appear below.

**Figure 3:**
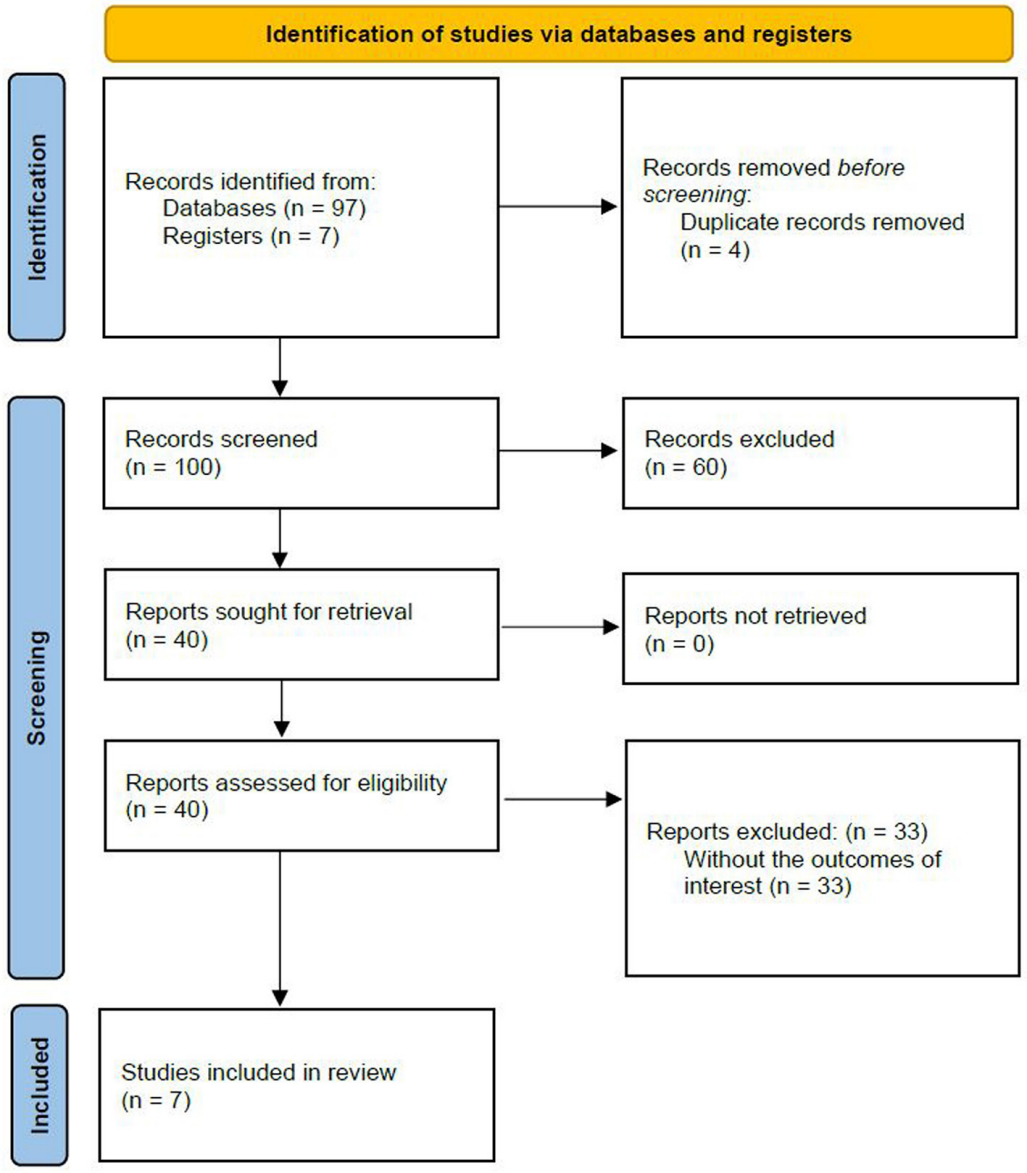
PRISMA diagram.

In the published article, there was also an error in Figure 4 as published. The image for Figure 4 should be the image that was published as Figure 3. The corrected Figure 4 and its caption appear below.

**Figure 4:**
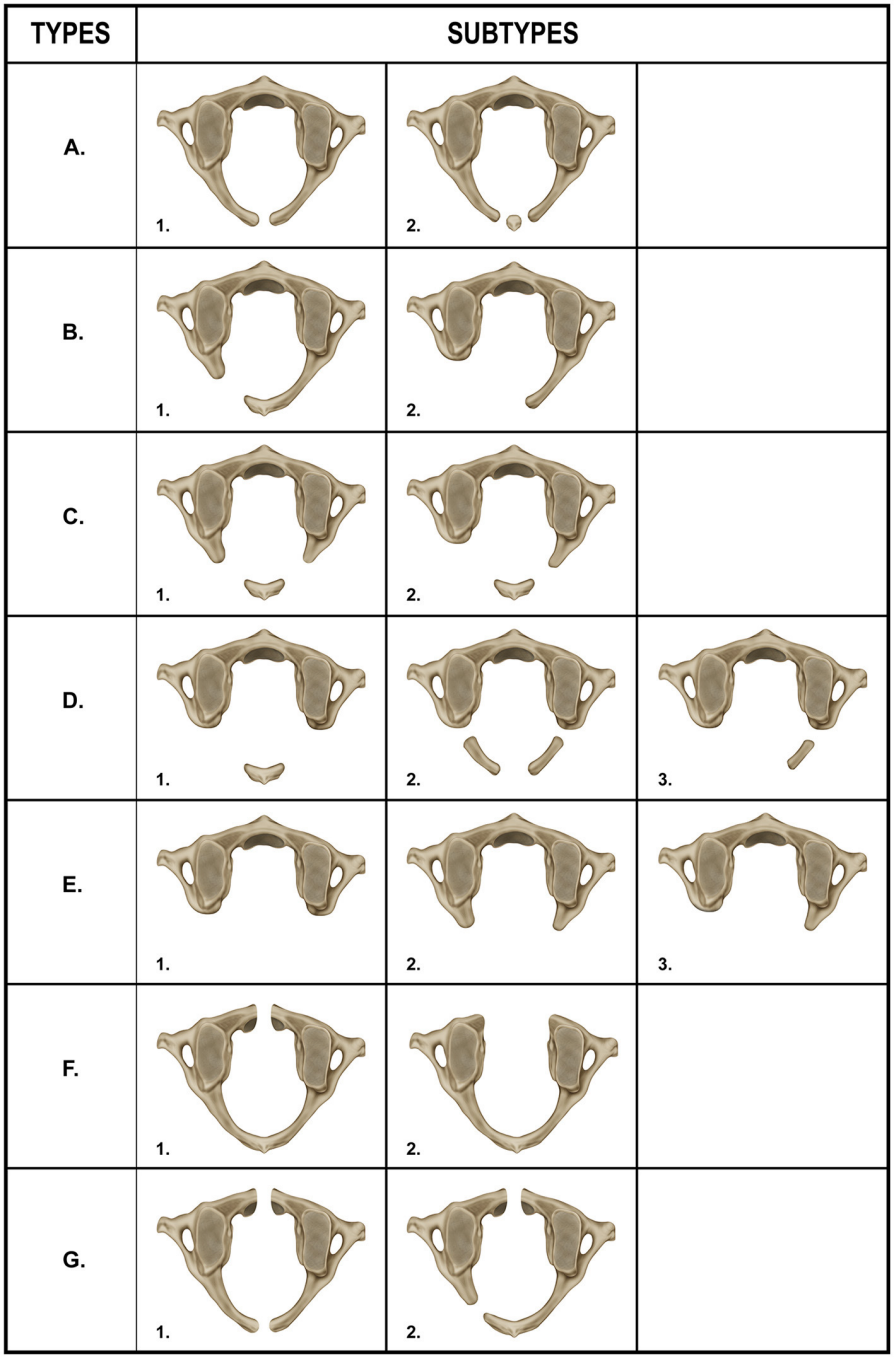
Recommended enhancements to the Currarino classification of atlas arch variations. Type A – 1 midline posterior cleft in the atlas, Type A – 2 small ossicle within the midline posterior cleft, Type B – 1 partial unilateral posterior cleft, Type B – 2 complete absence of one of the posterior hemiarches, and absence of the posterior tubercle, Type C – 1 bilateral partial defect in the posterior arch of the atlas with preservation of the posterior tubercle, Type C – 2 complete absence of one posterior hemiarch, partial defect in the other hemiarch, arm and posterior tubercle are preserved, Type D – 1 bilateral complete absence of both posterior hemiarches with a single posterior midline tubercle present, Type D – 2 bilateral cleft in both posterior arms and posterior tubercle absent, Type D – 3 unilateral absence of one posterior hemiarch, cleft of the contralateral arm and posterior tubercle absent, Type E – 1 complete absence of the posterior arch and absence of the posterior tubercle, Type E – 2 partial absence of the both posterior hemiarches and absence of the posterior tubercle, Type E – 3 absence of one of the posterior hemiarches, partial absence of the contralateral arm and posterior tubercle absent, Type F – 1 presence of a midline cleft in the anterior arch of the atlas, Type F – 2 complete absence of the anterior arch, Type G – 1 combined midline defects (clefts) in both the anterior and posterior arches of the atlas (bipartite atlas), Type G – 2 combined defects in the anterior and posterior arches (bipartite atlas) that include a midline cleft in the anterior arch and a unilateral cleft in one of the posterior hemiarches, the posterior tubercle is preserved.

In the published article, there was also an error in **Table 3** as published.

In row “Type D”, “2. A bilateral absence encompassing both posterior arms, coupled with the absence of the posterior tubercle. 3. The unilateral absence of one posterior hemiarch, along with the absence of the contralateral arm and the posterior tubercle.”, should be amended to “2. A bilateral cleft in both posterior hemiarches, coupled with the absence of the posterior tubercle. 3. The unilateral absence of one posterior hemiarch, cleft of the contralateral arm and cleft of the contralateral arm and absence of the posterior tubercle.”

In row “Type E”, “2. The absence of the posterior arch, while both posterior arms are preserved. In this configuration, the posterior tubercle is notably absent. 3. The absence of an entire posterior hemiarch, including the posterior tubercle, with the preservation of the contralateral arm.”, should be amended to “2. The partial absence of both posterior hemiarches, while both posterior arms are preserved. In this configuration, the posterior tubercle is notably absent. 3. The absence of an entire posterior hemiarch, including the posterior tubercle, with the partial preservation of the contralateral arm.”

In row “Type F”, “1. The presence of a cleft in the anterior arch of the atlas.”, should be amended to: “1. The presence of a midline cleft in the anterior arch of the atlas.”

In row “Type G”, “1. Combined defects in both the anterior and posterior arches of the atlas, characteristic of a bipartite atlas. This includes a cleft in the anterior arch and a midline cleft in the posterior arch. 2. Combined defects in the anterior and posterior arches, indicative of a bipartite atlas, encompassing a cleft in the anterior arch and a unilateral cleft in one of the posterior hemiarches, with the preservation of its arm and the posterior tubercle.”, should be amended to: “1. Combined defects in both the anterior and posterior arches of the atlas, characteristic of a bipartite atlas. This includes a midline cleft in the anterior arch, as well as a midline cleft in the posterior arch. 2. Combined defects in the anterior and posterior arches, indicative of a bipartite atlas, encompassing a midline cleft in the anterior arch and a unilateral cleft in one of the posterior hemiarches, with the preservation of the posterior tubercle.”

The corrected Table 3 and its caption appear below.

**Table 3:** Enhanced currarino classification: revised typology of atlas arches variations.

**Type A**	**1. The occurrence of a midline cleft in the atlas, which is attributed to the failure of the posterior midline fusion of the two hemiarches. 2. The presence of a small, distinct ossicle within the cleft, arising from the incomplete posterior midline fusion of the two hemiarches**.
**Type B**	1. The presence of a unilateral posterior cleft located in one of the arms of the posterior arch of the atlas. 2. The complete absence of one of the posterior hemiarches, encompassing the posterior tubercle.
**Type C**	1. A bilateral defect in the posterior arch of the atlas, characterized by the preservation of both posterior arms as well as the posterior tubercle. 2. The complete absence of one posterior hemiarch, coupled with a partial defect in the other hemiarch, yet maintaining the preservation of its arm and the posterior tubercle.
**Type D**	1. A bilateral and complete absence of both posterior hemiarches of the atlas. This anomaly is typically accompanied by a single, unattached posterior tubercle, often positioned in the midline. 2. A bilateral cleft in both posterior hemiarches, coupled with the absence of the posterior tubercle. 3. The unilateral absence of one posterior hemiarch, cleft of the contralateral arm and cleft of the contralateral arm and absence of the posterior tubercle.
**Type E**	1. The complete absence of the entire posterior arch of the atlas, which includes the absence of the posterior tubercle. 2. The partial absence of both posterior hemiarches, while both posterior arms are preserved. In this configuration, the posterior tubercle is notably absent. 3. The absence of an entire posterior hemiarch, including the posterior tubercle, with the partial preservation of the contralateral arm.
**Type F**	1. The presence of a midline cleft in the anterior arch of the atlas. 2. The complete absence of the entire anterior arch.
**Type G**	1. Combined defects in both the anterior and posterior arches of the atlas, characteristic of a bipartite atlas. This includes a midline cleft in the anterior arch, as well as a midline cleft in the posterior arch. 2. Combined defects in the anterior and posterior arches, indicative of a bipartite atlas, encompassing a midline cleft in the anterior arch and a unilateral cleft in one of the posterior hemiarches, with the preservation of the posterior tubercle.

In the published article, the Acknowledgment statement was mistakenly not included in the publication. The missing Acknowledgment statement appears below:

The authors apologize for these errors and state that this does not change the scientific conclusions of the article in any way. The original article has been updated.

